# Sensory and motor electrophysiological mapping of the cerebellum in humans

**DOI:** 10.1038/s41598-021-04220-9

**Published:** 2022-01-07

**Authors:** Reiko Ashida, Peter Walsh, Jonathan C. W. Brooks, Nadia L. Cerminara, Richard Apps, Richard J. Edwards

**Affiliations:** 1 Neurosurgery Department, Southmead Hospital, North Bristol Trust, Bristol, UK; 2 School of Physiology, Pharmacology and Neuroscience, University of Bristol, Bristol, UK; 3 Neurophysiology Department, Southmead Hospital, North Bristol Trust, Bristol, UK; 4 School of Psychology, University of East Anglia, Norwich, UK; 5 Neurosurgery Department, Bristol Royal Hospital for Children, University of Bristol NHS Foundation Trust, Bristol, UK; 6 Bristol Medical School, University of Bristol, Bristol, UK

**Keywords:** Neuroscience, Medical research, Neurology

## Abstract

Cerebellar damage during posterior fossa surgery in children can lead to ataxia and risk of cerebellar mutism syndrome. Compartmentalisation of sensorimotor and cognitive functions within the cerebellum have been demonstrated in animal electrophysiology and human imaging studies. Electrophysiological monitoring was carried out under general anaesthesia to assess the limb sensorimotor representation within the human cerebellum for assessment of neurophysiological integrity to reduce the incidence of surgical morbidities. Thirteen adult and paediatric patients undergoing posterior fossa surgery were recruited. Sensory evoked field potentials were recorded in response to mapping (n = 8) to electrical stimulation of limb nerves or muscles. For motor mapping (n = 5), electrical stimulation was applied to the surface of the cerebellum and evoked EMG responses were sought in facial and limb muscles. Sensory evoked potentials were found in two patients (25%). Responses were located on the surface of the right inferior posterior cerebellum to stimulation of the right leg in one patient, and on the left inferior posterior lobe in another patient to stimulation of left forearm. No evoked EMG responses were found for the motor mapping. The present study identifies challenges with using neurophysiological methods to map functional organization within the human cerebellum and considers ways to improve success.

## Introduction

The cerebellum is involved in the coordination of voluntary movements, postural balance and learning of new motor skills^1^. An increasing body of evidence indicates that the role of the cerebellum extends to cognitive functions^2–8^. Surgical damage to the cerebellum results in ataxia. In children, posterior fossa surgery can lead to cerebellar mutism syndrome in up to a third of patients, characterised by a transient loss of speech, behavioural impairments, emotional lability and hypotonia^9–11^.

Extensive anatomical and electrophysiological mapping studies in non-human species have shown that the cerebellum and its associated input/output pathways are functionally compartmentalised into modules^12–14^. In humans, magnetoencephalography (MEG) studies have reported short-latency responses (ca. 13–19 ms) in the cerebellum, evoked by median nerve stimulation^15,16^. As in other mammalian species, peripheral stimulation is therefore capable of synchronous activation of populations of neurons in the human cerebellum to generate substantial field potentials.

While non-invasive neurophysiological techniques have the temporal resolution to reveal such responses in humans, considerable averaging is required to detect cerebellar responses. Direct electrophysiological recording from the cerebellum overcomes this problem. To date, two studies have explored this possibility. Preliminary studies by Mottolese et al.^17,18^ reported evoked potentials in the posterior cerebellum (lobule VI), in response to stimulation of the hand and mouth muscles, while Hurlbert et al.^19^ also recorded evoked potentials from the posterior cerebellum in humans but in response to stimulation of the tibial nerve.

The present study extended these findings by exploring the possibility of recording field potentials from the surface of the human cerebellum evoked by upper or lower limb stimulation under general anaesthesia, as well as directly stimulating the cerebellar surface to determine if peripheral EMG responses can also be evoked. If either approach was successful, the findings could provide the basis for subsequent clinical application as a method to minimise damage to the sensorimotor cerebellar areas.

## Results

### Sensory mapping

**Figure 1:**
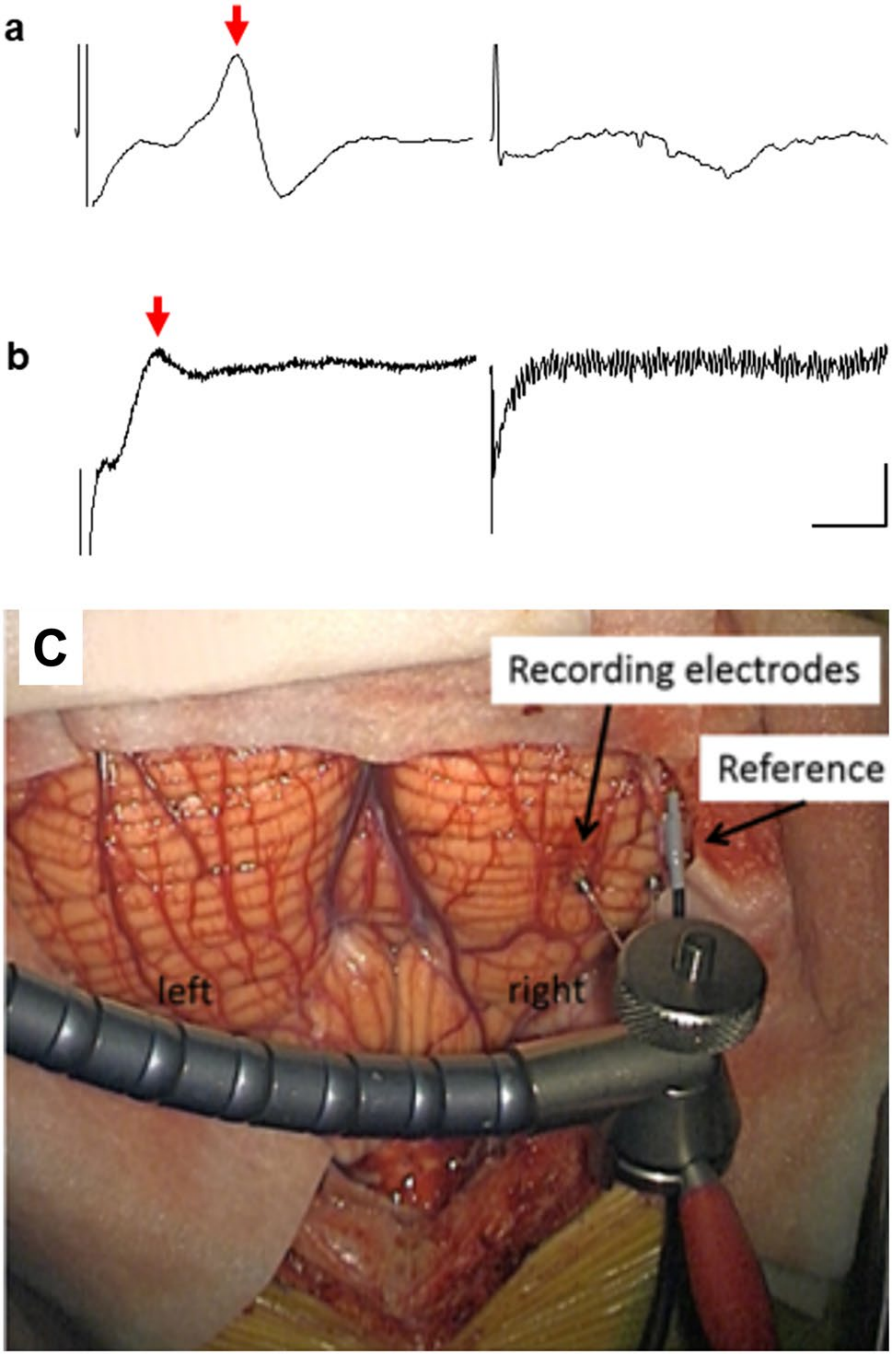
Cerebellar evoked potentials recorded from two patients. (**a**) Evoked cerebellar potential (arrow) recorded the right inferior posterior lobe in response to single pulse stimulation of the right tibial nerve from patient S2. The trace on the right shows lack of response from the same recording position following stimulation of the right median nerve. (**b**) Evoked cerebellar potential (arrow) recorded from the left posterior lobe from S6 in response to a train of stimuli (9 square pulses 0.5 ms, 10 ms interval, 2.7 Hz) delivered to the left forearm. The trace on the right shows no response to the same stimulus train from an adjacent cerebellar recording site. The trace from S2 is an average of 30 consecutive trials. The trace from S6 is an average of approximately 100 trials. Voltage scale bars = 10 µV in (**a**), 5 µV in (**b**); time base = 10 ms. (**c**) A photograph demonstrating the recording site in the right inferior posterior cerebellum from which an evoked potential was recorded from S2.

In eight patients, peripheral limb stimulation (cases S1–S8) evaluated if evoked field responses could be recorded from the cerebellar surface. Figure 1a shows an example evoked potential recorded from the right inferior posterior lobe of the cerebellum demonstrated in Fig. 1c (onset latency of 13 ms) in response to stimulation of the ipsilateral posterior tibial nerve in patient S2. Evoked potentials of a similar peak-to-peak amplitude (~ 16 μV) and onset latency were recorded at six adjacent recording positions on the cerebellar surface. By contrast, stimulation of the ipsilateral right median nerve at the same recording sites failed to evoke a detectable response (Fig. 1a).

**Figure 2:**
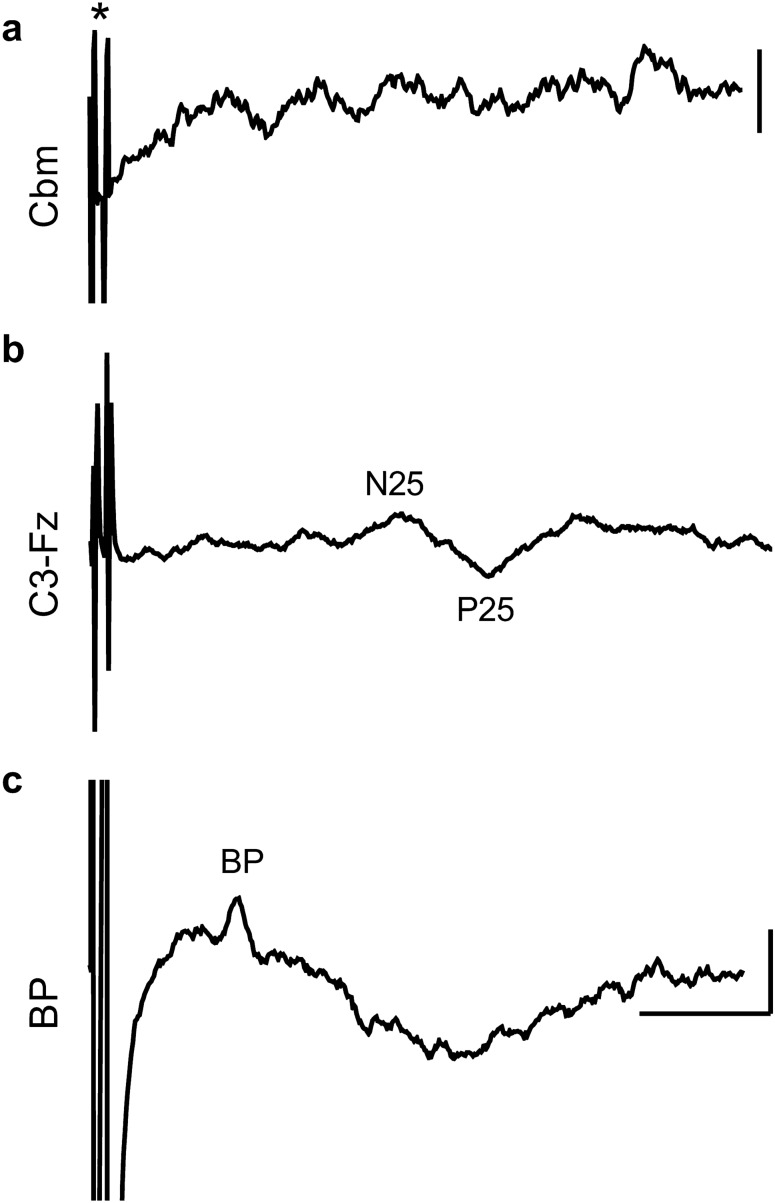
Example results from one patient (S4) with stimulation of the median nerve. (**a**) Stimulus artefact indicated by (*) but no detectable cerebellar (Cbm) response was evident. Average of 30 trials. (**b**) Cerebral SEPs (N25 and P25) recorded with a scalp electrode (C3) placed posterior to the central midline over the lateral parietal lobe, referenced to the Fz electrode placed over the frontal midline. (**c**) Brachial plexus (BP) peripheral nerve response. All SEP traces based on average of 50trials. Voltage scale bars = 10 µV in (**a–c**).

In a second patient (S6), stimulation of the extensor digitorum muscle in the ipsilateral left forearm evoked a response on the surface of the left inferior posterior lobe of the cerebellum (Fig. 1b, onset latency of ~ 11 ms, time interval between last stimulus in train and onset of response). The evoked potential was confined to one recording site; no responses were found in response to the same stimulus parameters applied to the right extensor digitorum. In the remaining six cases (S1, S3–5, S7–8) no detectable cerebellar responses could be found. In five cases (S3, S4, S5, S7, S8) the absence of a cerebellar response occurred despite the peripheral stimulation evoking a peripheral nerve volley and an SEP recorded over the contralateral parietal lobe (Fig. 2). The absence of any detectable cerebellar responses was therefore not likely due to the peripheral stimulation being ineffective in activating ascending sensory pathways.

### Motor mapping

**Figure 3:**
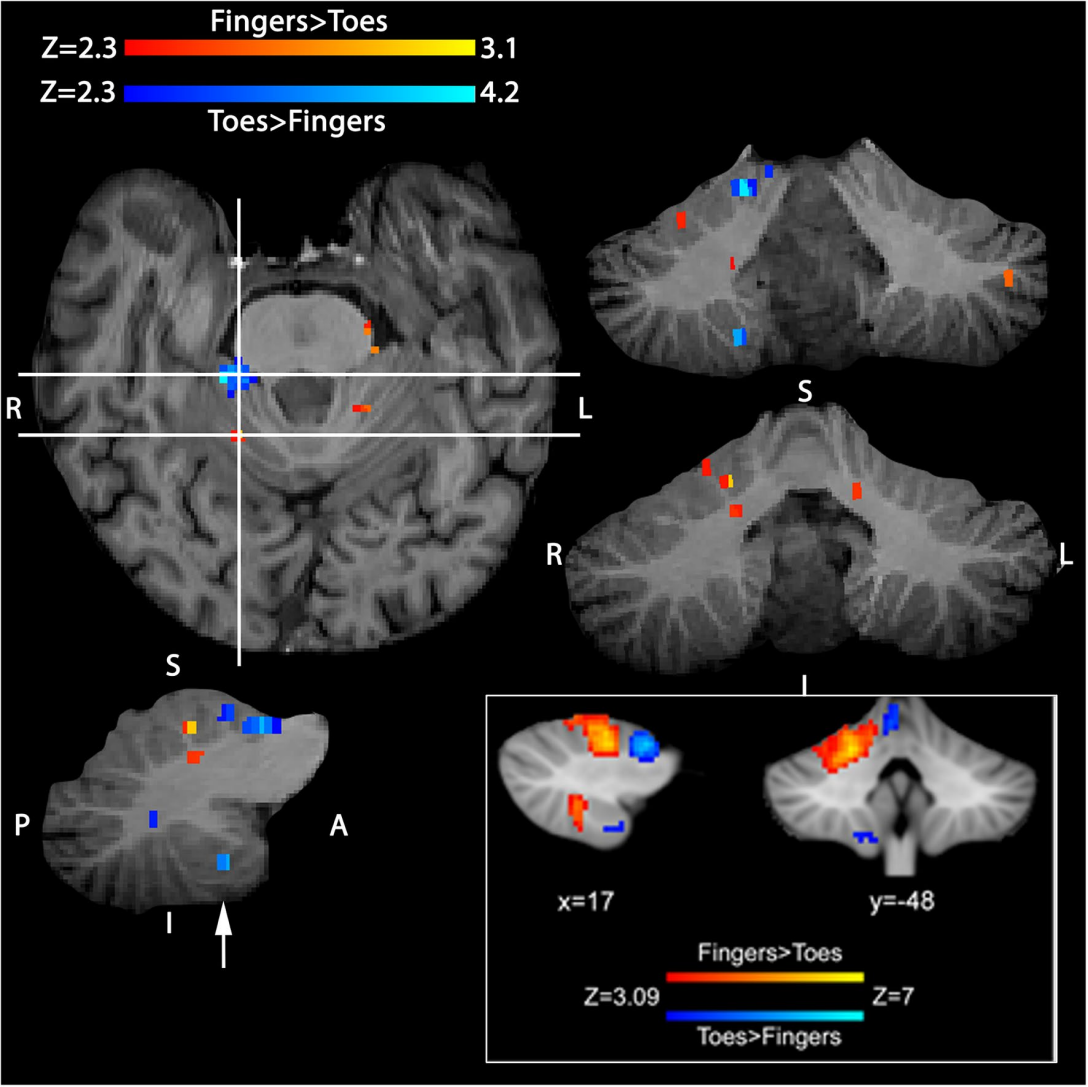
Pre-surgical cerebellar sensorimotor mapping with fMRI in a single patient (M4) undergoing posterior fossa ependymoma resection. Figure demonstrates two contrasts of sensorimotor activity (paced movement): (1) fingers greater than toes, shown in red/yellow, and (2) toes greater than fingers, shown in blue/light-blue on axial, coronal and sagittal sections, with an uncorrected significance threshold of P < 0.01 (i.e. Z > 2.3) superimposed on the subject’s high-resolution T1-weighted volumetric data. Horizontal lines on the axial slice demonstrate the y locations of coronal views to the right, vertical line indicates the x location of the sagittal slice immediately below. Note the activated region in the inferior posterior cerebellar lobe from the contrast toes greater than fingers, approximately 8 mm from the cerebellar surface shown with an arrow. Activity was primarily ipsilateral to the moved fingers/toes, and somatotopically arranged in the anterior lobe. No activity was seen in the posterior lobe for the fingers greater than toes contrast. For comparison, bottom inset shows group analysis results (data modified from^8^) demonstrating fingers and toes sensorimotor representation in the cerebellum in 20 healthy participants. The x and y coordinates of group activity are shown in the space of the Montreal Neurological Institute template (in millimetres)—with a cluster forming threshold of Z > 3.09 and corrected significance P < 0.05. Comparison to the two panels shows reasonable agreement between the single patient’s data and that obtained from the group of 20 healthy controls performing the same task.

Cerebellar stimulation was attempted in a further five patients (cases M1-M5). Cerebellar cortical stimulation did not result in any detectable EMG activity in the peripheral muscles recorded. In one patient (M4) pre-operative fMRI mapping produced BOLD activation in the inferior posterior lobe of the cerebellum—an area known to represent the motor function of ipsilateral toes (Fig. 3^8^). Despite this fMRI information to guide the cerebellar cortical stimulation sites, no peripheral muscle EMG responses could be found. The BOLD activation in the inferior posterior lobe was 8 mm deep from the stimulated cortical surface in the right cerebellar hemisphere.

## Discussion

We attempted to record evoked field potentials from the exposed surface of the human cerebellum in response to peripheral nerve stimulation in eight patients; and in a further five patients we attempted to record evoked EMG responses to direct stimulation of the cerebellar surface. This was performed during the scheduled intra-operative break taken by the surgeon, limiting data collection to approximately thirty minutes, allowing either sensory or motor mapping to be carried out in each patient. Time constraints precluded the monitoring of both modalities in an individual patient. Only peripheral stimulation was successful in evoking responses, and this was limited to two out of eight patients. In both successful cases the responses were recorded on the surface of the inferior posterior cerebellar hemisphere (Fig. 1c for S2), ipsilateral to the site of limb stimulation. The first case (S2) had a lesion in the right hemisphere extending into the vermis. The second case (S6) had a vermal lesion.

We have considered a number of factors that may have contributed to the low success rate for recording responses. The subjects evaluated all had a symptomatic disease process (most commonly tumour) affecting the cerebellum that may have affected signal transmission through either anatomical neuronal pathway damage, anatomical distortion or peri-tumoural oedema. Whilst tumour location varied the surgical exposure was relatively consistent in terms of the posterior lobe exposure as a wide exposure helps limit the amount of intra-operative retraction required, so lack of access to “recordable” exposed cerebellar hemisphere was not a factor, further in three cases an extensive area of anterior lobe was also accessible. However, given the small sample size it is difficult to be conclusive about the extent to which cerebellar disease affected our findings although from a practical standpoint, intra-operative monitoring would only have clinical utility if recordings are reproducible in the disease state.

fMRI studies would suggest that limb representation may be more extensive in this region of the cerebellum and therefore easier to locate^8^, we therefore considered whether lack of access to the anterior cerebellum may have been a factor to the lack of recorded responses. In the majority of cases a wide bilateral exposure was undertaken, allowing similar areas of posterolateral hemisphere to be available for testing. Two of the motor mapping patients (M2 and M3) and one sensory mapping patient (S3) had, in addition, generous access to the anterior cerebellar lobe and this did not increase the success rate of obtaining responses. It therefore seems unlikely that this can fully explain our negative results. We also considered whether the number of trials used for averaging could have contributed to the failure to record responses—however responses were obtained when 30 trials were used as well as when 100 trials so fewer trials did not seem to be a major factor.

In an attempt to increase the success rate of recording cerebellar evoked responses, a number of changes were made to the peripheral stimulation protocol. For example, paired pulse stimulation is known to facilitate cerebellar responses^20^. And similar peripheral stimulus parameters to those used by Mottolese and colleagues (personal communication) to evoke potentials in the human cerebellum were also attempted. With the caveat of not being able to draw firm conclusions from a small sample size, it was not evident that any of these changes significantly improved our success rate (1 in 4). The finding in one patient that BOLD activation was located below the cerebellar cortical surface (consistent with a previous fMRI report^8^) may have attenuated the strength of the stimulus and impacted on the response. Finally, the effect of anaesthetic on transmission in spino-cerebellar pathways may be important factors to explain our relatively low yield of results, as anaesthetics are known to have a profoundly depressing effect on such pathways^21–23^.

Cerebellar cortical stimulation in five additional patients did not demonstrate any EMG responses. Various stimulation parameters previously used in animal studies^24^ were tested in addition to standard parameters used for transcranial MEP monitoring. Our failure to evoke EMG responses contrasts with those of Mottolese et al.^17^ who reported evoked EMG responses in humans as a result of cerebellar surface stimulation, although in their study only 8% of cerebellar stimulation sites evoked a detectable EMG response. Mottolese et al.^17^ also used biphasic stimulation whilst this may increase the overall electric charge delivered to the cerebellum but there is not persuasive evidence that this increased the overall yield of response points in their study compared to this one A preliminary study^25^ suggests an alternative future strategy might be to monitor the indirect effect of cerebellar stimulation on motor output by studying transcranial motor evoked potentials.

Another important consideration is that our study was limited to assessing sensorimotor pathways. Cognitive function including language function, which may be more relevant to cerebellar mutism syndrome, could not be assessed in the anaesthetised subject. Moreover, in terms of anatomy, damage to the superior cerebellar peduncle or the dentatothalamic cortical pathway is most likely to lead to cerebellar mutism syndrome. These structures were not accessible from surface transcranial direct current stimulation. One way of assessing language function is to use single-pulse transmagnetic stimulation (spTMS) which has a more focused and restricted spatial extent of stimulation. Such an approach allows stimulation of language areas in the cerebral cortex such as Wernicke’s area to assess the presence of cerebellar evoked responses^26^, but this approach would also require technique outside of the ethical approval obtained in this study.

Given that previous fMRI studies^6–8^, have successfully mapped sensorimotor, language and verbal working memory functions of the cerebellum, demonstrating compartmentalized representation, it might be argued that fMRI would be a more useful approach. Indeed, frequency maps have found that while localization of sensorimotor representation is similar between individuals, localization is more variable for cognitive representation^8^. Individualised pre-operative fMRI mapping could therefore aid surgical planning. It should however be noted that fMRI mapping through the blood oxygenation level dependent (BOLD) response is an indirect marker of neuronal activity, and currently does not allow real time monitoring during surgery. Pre-operative surgical guidance may also be limited due to brain shift following dural opening, which distorts brain anatomy.

The waveform and onset latencies to upper limb stimulation recorded under anaesthesia in the present study are consistent with those obtained using MEG in awake humans^15,16^ and those obtained from animal studies. For example, in the awake cat, cerebellar cortical responses identified as climbing fibre in origin and evoked by stimulation of the superficial radial nerve in the ipsilateral forelimb can be evoked in the anterior (lobule V) and posterior lobes (lobule VII, rostral paramedian lobule) with an onset latency ranging between 9 and 14 ms^27,28^. This is similar to the onset latency for climbing fibre responses reported in anaesthetised cats, rats and ferrets^20,29–31^. By comparison, responses in animal experiments attributable to activation of spino-cerebellar pathways terminating as mossy fibres have an onset latency of ~ 5 ms^21,32,33^ Taken together this suggests that spino-cerebellar pathways can be activated in a range of species by upper limb stimulation. Assuming roughly similar conduction velocities in the ascending tracts and given the longer conduction distances in human, this suggests that the cerebellar responses in human may be mainly mossy fibre in origin.

Consistent with this interpretation are our results from lower limb stimulation. In the present study under anaesthesia, the onset latency of these responses were ~ 12 ms. By comparison, the onset latencies of climbing fibre responses evoked by hindlimb stimulation and recorded in homologous regions of the posterior lobe of the cerebellum in anaesthetised rats are longer at about 16–19 ms^20^. Unless spino-olivocerebellar pathways in human are much faster in conduction than in other species, this suggests that the responses recorded in the present report are mainly mossy fibre in origin, although Purkinje cell recording would be required to test this directly.

In conclusion, the present study demonstrates it is possible to record under anaesthesia responses from the surface of the human cerebellum evoked by peripheral stimulation. The advantages of intraoperative neurophysiological monitoring of the cerebellum include facilitation of real-time, individualised monitoring to optimise neurological outcome. It may trigger a surgical stop point, for example by detecting attenuation of evoked potentials. The challenge was that the potentials were not consistently found, limiting their use for intraoperative neurophysiological monitoring to preserve cerebellar function intra-operatively. A more extensive study would be required to optimize stimulation and recording parameters before such an approach could be used intra-operatively to reliably monitor cerebellar somatosensory and motor function.

## Methods

### Patients

Study approval was obtained by the Frenchay Research Ethics Committee. Informed consent was obtained from adult patients. Informed consent was obtained from the legal guardians or parents for paediatric patients. The study was conducted in accordance with the Declaration of Helsinki. Any patient from Frenchay Hospital, Southmead Hospital and Bristol Royal Hospital for Children undergoing a posterior fossa craniotomy between 2012 and 2017, over the age of two, fluent in English, total operation duration of more than three hours and those without any contraindications to neurophysiological monitoring were included in the study. Patients with previous history of posterior fossa craniotomy and with any pre-existing neurological conditions were excluded from recruitment.

Thirteen patients (nine male) were recruited in total. Age range was 3–63 years (median age 24 years). Eight patients underwent peripheral electrical stimulation and sensory mapping of the cerebellum. Five patients underwent cerebellar cortical electrical stimulation for motor mapping.

### Surgery

**Figure 4:**
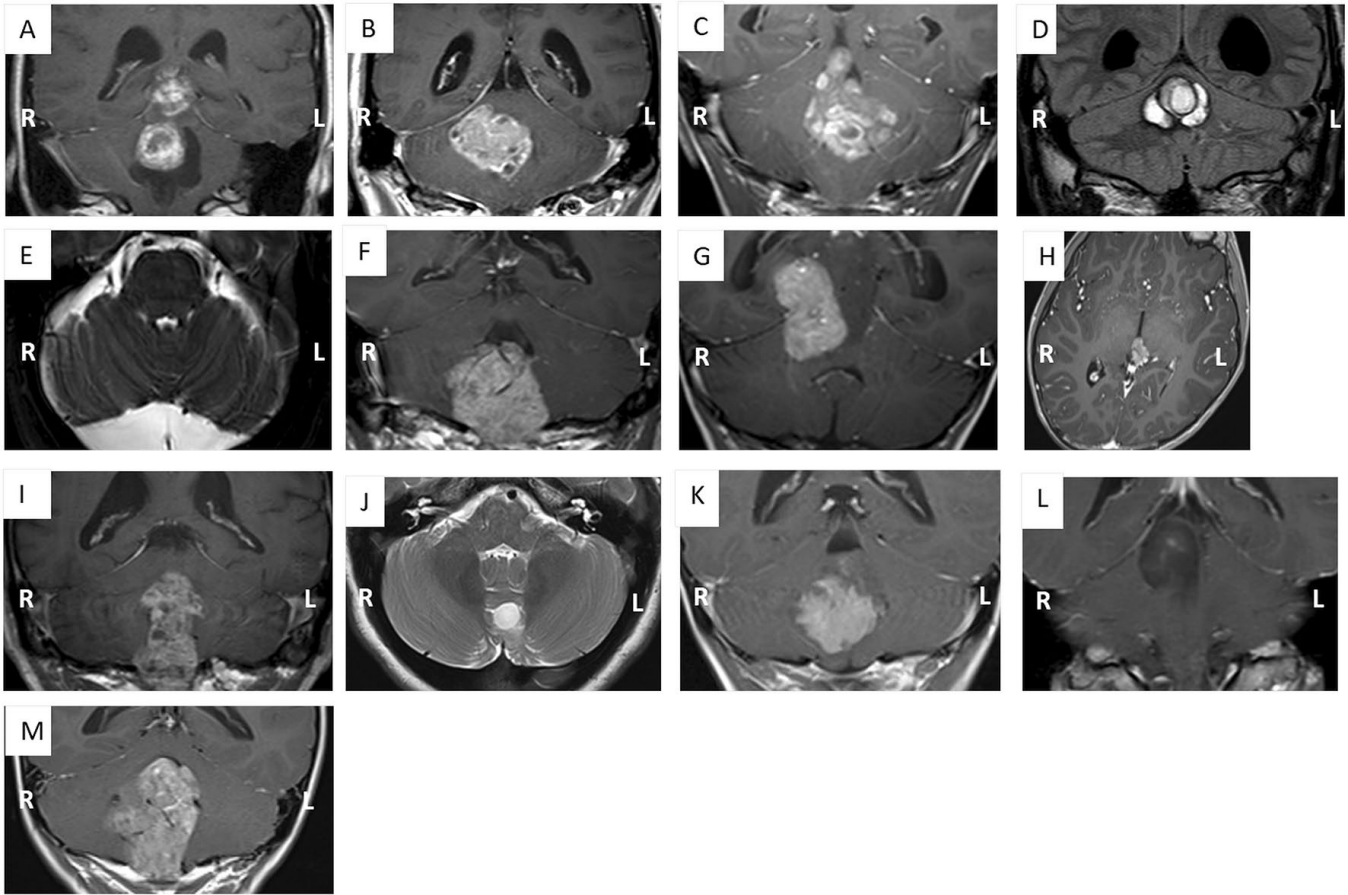
MRI scans demonstrating the pathologies of participants. (**A**) S1 ganglioglioma, (**B**) S2 metastasis, (**C**) S3 ependymoma, (**D**) S4 cavernoma, (**E**) S5 supracerebellar arachnoid cyst, (**F**) M1 ependymoma, (**G**) M2 pineoblastoma, (**H**) M3 pineoblastoma, (**I**) M4 ependymoma, (**J**) S6 pilocytic astrocytoma, (**K**) S7 ependymoma, (**L**) S8 diffuse astrocytoma, (**M**) M5 ependymoma.

Pathologies of patients are demonstrated in Fig. 4. Surgery was performed under propofol anaesthesia to minimise interference with neurophysiological monitoring^34^. A midline posterior fossa craniotomy approach was used for all patients. Patients were registered to a neuro navigation system by surface registration to the uploaded T1 fine cut axial MRI with gadolinium.

Standard intraoperative monitoring of motor and sensory evoked potentials (MEPs and SEPs) and cranial nerve activity allowed monitoring of the corticospinal tract and somatosensory pathways and the afferent nerve volley. EMG recordings using twisted pair needle electrodes (Ambu, Copenhagen, Denmark) were obtained from the muscles of the face, oropharynx and shoulders supplied by the cranial nerves, small muscles of the hands (abductor pollicis brevis, the adductor digiti minimi or the 1st dorsal interosseous) and abductor hallucis brevis^35^. SEP recordings were obtained from scalp using corkscrew electrodes (Ambu Copenhagen, Denmark) placed over the contralateral parietal lobe^35^, in response to the stimulation of the median nerve (upper limb) and posterior tibial nerve (lower limb) (Ambu disposable Neuroline stick on electrodes). Cerebellar evoked potentials were recorded from the cerebellar cortical surface (Fig. 1C), prior to the tumour resection. Corkscrew stimulation electrodes (Ambu, Copenhagen, Denmark) were positioned on the scalp overlying the precentral gyrus for monitoring MEPs in the small muscles of the hands and in abductor hallucis brevis.

### Limb stimulation

Peripheral stimuli were delivered by a 32 channel Neuromaster IOM system (Nihon Kohden, Tokyo, Japan). Stimulation parameters were: in S1, single pulse constant current (0.2 ms duration), 5.1 Hz upper limb and 3.1 Hz lower limb (identical to the stimulation parameters used for standard clinical SEP monitoring in patients)^36^. Or in S2-8, based on those used in animal studies with stimulus rates from 0.2 to 0.5 Hz^20,37^. For cases S4 and S5, paired pulse stimulation was delivered (1 ms inter-stimulus interval, ISI). The peripheral stimulus intensity was adjusted to evoke a small but detectable twitch in the corresponding body part. This was approximately 20 mA for the arms and 30–40 mA for the legs.

Three patients (S6-8) underwent sensory mapping of the cerebellum using stimulus parameters based on the study performed by Mottolese et al.^18^ (Table 1, nine pulses, 0.5 ms duration, ISI 10 ms, 2.7 Hz, at an intensity which produced a muscle twitch). Using these parameters, in one patient (S6) the forearm extensors and the tibialis anterior muscles in the lower leg were stimulated using twisted pair needle electrodes (Ambu, Copenhagen, Denmark), in addition to median and posterior tibial nerves. More proximal limb muscles, biceps and quadriceps were stimulated for the two other patients (S7, S8). Table 1 indicates the stimulus parameters and limb stimulation sites used for all eight sensory mapping patients.

**Table 1:** The summary of recruited patient demographic and stimulation parameters for sensory mapping.

Case no.	Age	Sex	Histology	Location of pathology	Stimulation location	Peripheral stimulation parameters
S1	47	F	Ganglioglioma	Tectum IVth ventricle	Median and posterior tibial nerves	Single 0.2 ms square pulse, 5.1 Hz, posterior tibial nerve Single 0.2 ms square pulse 3.1 Hz, median nerve
S2	49	M	Metastasis unknown primary	Right Cerebellar hemisphere	Median and posterior tibial nerves	Single 0.2 ms square pulse, 0.5 Hz
S3	7	M	Ependymoma	IVth ventricle	Median and posterior tibial nerves	Single 0.2 ms square pulse, 0.5 Hz
S4	27	M	Cavernoma	Cerebellar vermis	Median and posterior tibial nerves	Paired 0.2 ms square pulses, 1 ms interval at 0.5 Hz
S5	39	M	Arachnoid cyst	Supra cerebellar	Median and posterior tibial nerves	Paired 0.2 ms square pulses, 1 ms interval at 0.5 Hz
S6	16	F	Pilocytic astrocytoma	Cerebellar vermis	Lower arm, lower leg	9 square pulses 0.5 ms, 10 ms interval, 2.7 Hz
S7	6	M	Ependymoma	IVth ventricle	Upper arm, thigh	9 square pulses 0.5 ms, 10 ms interval, 2.7 Hz
S8	24	M	Diffuse astrocytoma	Brainstem	Upper arm and thigh	9 square pulses 0.5 ms, 10 ms interval, 2.7 Hz

### Sensory mapping: cerebellar evoked potentials

Electrophysiological recording from the exposed surface of the cerebellum was carried out prior to tumour resection in the eight sensory mapping patients (Table 1). A bipolar 2 mm ball tipped stimulation probe (Inomed, Emmendingen, Germany) with 5–8 mm width between the two contact points was used for seven of the eight patients (S1-4, S6-7). Electrophysiological signals were recorded differentially between one of the contact points and an indifferent electrode placed nearby in the subcutaneous tissue alongside the incision. The data were amplified (×1000) and bandpass filtered (30 Hz to 3 kHz). The probe was held free hand for the first patient (S1) and gently placed at different positions on the cerebellar cortical surface. For the remaining patients (S2–S7), the probe was fixed in a flexible arm retractor to minimise movement artefacts during recording. In all cases, the bipolar probe was moved in increments of approximately 5 mm laterally and rostro-caudally in a systematic manner to cover the entire exposed cerebellar surface. A four-contact recording strip with 10 mm spacing (Ad-tech Medical Instrument Corporation, Oak Creek, USA) was used in one patient (S5). SEP recordings provided a positive control that the peripheral stimulation was effective in generating an ascending sensory volley (Fig. 2).

### Motor mapping: cerebellar stimulation

In five patients (M1-5) the cortical surface of the cerebellum was stimulated using a monopolar probe in order to evoke EMG responses from the nasalis and orbicularis oris, biceps, forearm (extensor digitorum communis and flexor carpi radialis), small hand muscles, quadriceps, tibialis anterior and abductor hallucis, using parameters based on human intraoperative transcranial MEP monitoring and animal and human cerebellar stimulation (Table 2^17,38^).

**Table 2:** The summary of recruited patient demographic, stimulation parameters for motor mapping.

Case no.	Age	Sex	Histology	Location of pathology	Cerebellar stimulation parameters
M1	55	M	Ependymoma	IVth ventricle	5 pulses, pulse duration 0.3 ms, 400 Hz, 10, 20, 30 mA, 11.5 ms total stim duration
M2	3	F	Pineoblastoma	Pineal	5 pulses, pulse duration 0.3 ms, 400 Hz, 10, 20, 30 mA, 11.5 ms total stim duration
M3	11	M	Pineoblastoma	Pineal	35 pulses, 150 Hz, pulse duration 0.3 ms, 10 mA, 235 ms total stim duration,
M4	63	M	Ependymoma	IVth ventricle	35 pulses, 150 Hz, pulse duration 0.3 ms, 10 mA 235 ms total stim duration,
M5	6	F	Ependymoma	IVth ventricle	35 pulses, 150 Hz, pulse duration 0.3 ms, 10 mA 235 ms total stim duration,

Stimulation parameters were: (1) five anodal square wave pulses, duration 0.3 ms, ISI 2.5 ms, 10–30 mA in two patients (M1-2) or (2) in three patients (M3-5), 35 anodal square pulses, duration of 0.3 ms, ISI 2.5 ms, 10 mA, total stimulation duration of 235 ms. Bipolar stimulation was also carried out in one patient (M3) using the same bipolar probe. Cathodal stimulation was carried out in one patient (M2, see Table 2). Charge density ranged from 0.7 to 4.5 micro C/cm2/phase. This was below the maximum safe charge density (up to 7.4 micro C/cm2/phase) based on human chronic cerebellar stimulation and non-human primate cerebellar stimulation^39,40^.

### Data analysis

Data were analysed offline using Spike2 software (CED, Cambridge, UK). Approximately 30 trials were averaged per recording site for six sensory mapping patients (S1-5, S8). For the two remaining sensory mapping patients (S6,7) 100 to 150 trials were averaged per recording site. Recordings from every cerebellar site for the sensory mapping cases, and the EMG recording from peripheral muscles for the motor mapping were carefully examined for evoked potentials at the time of recording. If a response was evident, then average onset latency, which was taken from the first stimulus pulse, and peak-to-peak amplitude were measured offline.

### Functional MRI

In one motor mapping patient (63 year-old male, M4) with a fourth ventricular ependymoma, pre-operative functional MRI was undertaken in order to increase the likelihood of locating sites for cerebellar stimulation to evoke a peripheral response. The sensorimotor fMRI paradigm involved the patient moving their fingers or toes at an irregular rhythm directed by the flashing words ‘fingers’ or ‘toes’ on an LCD screen^8^. fMRI data were analysed using the FSL software package (http://fsl.fmrib.ox.ac.uk). The functional data were uploaded onto the Stealth navigation system (Medtronic, Minneapolis, USA). The fMRI data and the T1 structural scan (Magnetization-Prepared Rapid Gradient-Echo sequence, MPRAGE^41^) were in NIfTI (Neuroimaging Informatics Technology Initiative) format. These were converted to DICOM (Digital Imaging and Communications in Medicine) format compatible to the Stealth navigation system. These were then transformed onto the subject’s T1 structural MRI scan. Cerebellar surface stimulation was carried out at the closest, accessible surface site from the BOLD activated area within the cerebellum.
